# Likes and impulsivity: Investigating the relationship between actual smartphone use and delay discounting

**DOI:** 10.1371/journal.pone.0241383

**Published:** 2020-11-18

**Authors:** Tim Schulz van Endert, Peter N. C. Mohr

**Affiliations:** 1 School of Business and Economics, Freie Universität, Berlin, Germany; 2 WZB Berlin Social Science Center, Berlin, Germany; 3 Center for Cognitive Neuroscience Berlin, Freie Universität, Berlin, Germany; Shandong University of Science and Technology, CHINA

## Abstract

The omnipresence of smartphones among adolescents and adults gives rise to the questions about excessive use and personality factors which are associated with heavier engagement with these devices. Previous studies have found behavioral similarities between smartphone use and maladaptive behaviors (e.g. drinking, gambling, drug abuse) in the context of intertemporal choice but mostly relied on participants’ self-reports regarding engagement with their phone. In this study, we collected actual usage data by smartphone application from 101 participants and assessed their tendency to discount future rewards, their reward responsiveness, self-control and consideration of future consequences. We found that smartphone screen time was correlated with choosing smaller immediate over larger delayed rewards and that usage of social media and gaming apps predicted delay discounting. Additionally, smartphone use was negatively correlated with self-control but not correlated with consideration of future consequences. Neither psychological variable could mediate the relationship between smartphone usage and delay discounting. Our findings provide further evidence that smartphone use and impulsive decision-making go hand in hand and that engagement with these devices needs to be critically examined by researchers to guide prudent behavior.

## Introduction

For most people in the developed world the smartphone has become a constant companion. Recent surveys estimate that 76% of adults in advanced economies own a smartphone [1] while the penetration among adolescents has reached more than 80% [2]. Depending on the geography of the sample and the research methodology, the average duration for which smartphone owners are actively engaged with their devices ranges from 4.7 hours [3] to 8.8 hours per day [4]. Furthermore, more than 33% of smartphone users report that they access their smartphones within the first five minutes of waking up in the morning and more than 40% check their phone during the night [5].

These statistics have naturally given rise to the question about excessive use of smartphones and its implications. Frequent notifications, immediate access to information and social feedback may make it difficult to refrain from engaging with the device, even if it is inappropriate or even dangerous (e.g. while driving) to do so [6]. While the concept of smartphone addiction is not yet included in the Diagnostic and Statistical Manual of Mental Disorders, Fifth Edition (DSM-5), several authors have found significant overlap between excessive smartphone use and substance-related disorders defined in the DSM-5 [7, 8]. Approaching the issue from a different angle, cognitive scientists have started to look at individual differences in functions such as attention [9], memory [10] and decision-making [11] and their relationships with smartphone use. Due to the relative novelty of smartphones, this research field is still in its infancy but dynamically growing [6]. One strand of the literature, which investigates the association between smartphone usage and impulsive choice, i.e. an individual’s preference for smaller, immediate rewards over larger, delayed rewards has been a particularly fruitful avenue for research.

Human and non-human animals typically discount rewards as a function of the delay of their delivery, implying that a reward received today is worth more than the same reward received at a later point in time [12]. This tendency is referred to as delay discounting and is revealed in intertemporal choice problems, where participants are faced with the tradeoff between the delay and the amount of a reward (e.g. choosing 50€ today or 55€ in one week). Delay discounting has been studied thoroughly in the past decades, resulting in the emergence of two main models which seek to capture intertemporal choice behavior: exponential and hyperbolic discounting, the latter providing a better fit to the majority of empirical data [12]. The corresponding equation V = A / (1+kD) (V is the present value of the future reward, A is the reward amount and D is the delay to the reward) contains one free parameter k, which represents an individual’s discount rate. The larger this discounting parameter, the more the individual devalues remote rewards and is therefore relatively more impulsive than a person with a lower discount rate. Individuals’ discount rates have been shown to be relatively stable over time, which is why delay discounting is widely considered to be similar to a personality trait [13, 14]. Furthermore, delay discounting has been associated with a host of maladaptive behaviors, such as drug abuse [15] problematic drinking [16] and gambling [17]. Studies have repeatedly shown that addicts discount future rewards more extremely than control participants, making the degree of delay discounting a reliable indicator for addictions of different nature [14].

While the concept of smartphone addiction is still under debate, researchers agree that impulsive decision-making as revealed in delay discounting paradigms is a relevant factor in the context of smartphone use [18, 19]. On the one hand, there is substantial evidence that social media, messaging and gaming—the most popular activities on smartphones [20–24]–are characterized by activation of primarily reward-related brain regions [25–29], highlighting the central role that gratification plays in engagement with the smartphone. On the other hand, excessive smartphone use has also been shown to have a negative impact on important parameters, such as sleep quality [30], stress levels [31], academic success [32] or overall well-being [33]. Thus, high smartphone users implicitly face a trade-off between gratification in the present and adverse consequences in the future.

First studies have indicated a positive association between smartphone usage and the discounting of future rewards. Wilmer and Chein [18] found that heavier engagement with the phone was positively correlated with an individual’s discounting rate and greater impulsivity, the latter being assessed via a questionnaire and a behavioral measure. Similarly, Tang et al. [19] found that high and medium smartphone users in their sample more often chose a smaller immediate reward than low users and showed a bias in evaluating the time and monetary value dimension within an intertemporal choice task. In another study focusing on one aspect of smartphone usage, Delaney et al. [34] found that Facebook addicts discounted future rewards more heavily than matched controls. In a comprehensive field experiment Hadar et al. [35] compared a group of heavy smartphone users, as determined by questionnaires and verified by usage data recorded over a seven-day period, to a group lacking any experience with smartphones on a range of behavioral measures. Among other cognitive differences, they found that the heavy users behaved more impulsively within a delay discounting paradigm than the non-users. Additionally, to allow for causal claims regarding behavioral and neural changes associated with three-month smartphone exposure the authors also compared non-smartphone users to participants who received smartphones for the first time. The authors, however, could not observe an effect of smartphone usage on delay discounting.

These studies have provided initial valuable insights about the relationship between smartphone usage and delay discounting. However, they also exhibit two limitations, which may on the one hand challenge the reliability of the results and on the other hand restrict the conclusions which can be drawn from their findings. The first limitation concerns the measurement method of smartphone usage; previous studies have mostly relied on participants’ self-reports regarding the patterns of smartphone engagement. Typically, questionnaires such as the Smartphone Addiction Scale [36] or the Smartphone Addiction Inventory [7] are used to group participants into heavy and low users. While these scales have been proven to reliably measure smartphone addiction, they are based on subjective statements (e.g. “My life would be empty without my smartphone”) rather than on objective criteria such as screen time or checking behavior and therefore may not be the most suitable instruments to measure engagement with the smartphone. In a few instances, participants are directly asked to estimate how much time they spend with smartphone apps or how often they check their phones. Recent studies have shown that these kinds of self-reports are often unreliable due to participants’ limited capacity in correctly estimating engagement with their phones. Kobayashi and Boase [37] found that Japanese phone users overestimated the number of calls made and text messages sent. Similarly, Boase and Ling [38] concluded that self-reports about calls and text messages correlated only moderately with actual log data of their large Norwegian sample. Andrews et al. [39] came up with similar results, finding that the estimated number of times an individual used her phone on a typical day did not correlate with actual usage and that neither estimated nor actual usage was related to scores on the Mobile Phone Problem Use Scale [40]. As an exception, Hadar et al. [35] also recorded actual usage data by means of an application, which was installed on participants’ smartphones at the beginning of their experiment. While this enabled the authors to verify their initial questionnaire-based grouping of participants into high and low users, participants became aware that their usage was being observed. This awareness may on the one hand affect participants’ natural smartphone-related behavior and on the other hand change the way participants behave in tasks aimed at measuring the effects of smartphone usage [6].

The second limitation is constituted by the scope of the assessment of smartphone usage. Either this variable is assessed broadly (i.e. overall engagement with the device, without regard of the specific apps/functionalities used) or narrowly by focusing on one out of the many aspects of smartphone engagement, such as social media. Both approaches do not allow for the identification of drivers of the relationship between smartphone engagement and delay discounting. However, a novel method leveraging the native Apple iOS feature “battery usage” has made it possible to overcome the above-mentioned measurement issues [23]. For this, researchers collect data from participants’ iPhones which show the exact duration of all applications used recently. In addition to providing a comprehensive picture of individuals’ usage patterns, this method is also non-intrusive as participants are not aware that their usage data are collected, as opposed to e.g. installing an app which records usage data and thereby potentially influencing naturalistic behavior.

This method has already been employed successfully when relating smartphone usage to other variables, such as well-being [22].

Furthermore, a promising neuroscientific model has emerged recently, which seeks to explain the mechanisms underlying intertemporal choice. According to this model, the variability in people’s tendency to discount delayed rewards may be explained by individual differences in reward valuation, cognitive control and the ability to imagine future outcomes of decisions (prospection) [14]. Investigating these three personal dispositions and the nature of their relationships with smartphone usage as well as delay discounting may further our understanding of the variables associated with excessive use.

Thus, to replicate and extend previous findings on the relationship between smartphone usage and delay discounting, this study investigates the following two hypotheses:

Hypothesis 1: Actual smartphone usage is positively correlated with the tendency to discount future rewards (delay discounting).
Hypothesis 2: The relationship between smartphone usage and delay discounting is mediated by reward valuation, cognitive control and prospection.

We collected, in addition to self-reports, actual usage data by application from a sample, which is characterized by widespread smartphone ownership. In parallel, we elicited delay discounting with a widely used intertemporal choice paradigm along with personal dispositions. Our study contributes to the literature by showing a relationship between delay discounting and smartphone usage based on actual usage data, by uncovering two app categories which predict delay discounting and by demonstrating a link between self-control and smartphone usage.

## Methods

### Participants

116 participants (53% female, mean age 22 years) were recruited from the volunteer database of the Berlin Social Science Center (WZB) using the software ORSEE [41]. Six participants declined to provide their phone usage data upon arrival at the experiment, but took part in all other parts of the study. For five participants the usage data was not available due to an outdated or malfunctional operating system. Usage data of another four participants were not usable, since they brought a spare or a borrowed phone to the experiment. Net of these data points, data from 101 participants (52% female) were included in the analysis.

### Measures

#### Net screen time

To assess how long and for which activities participants used their smartphone, data provided by the iOS feature “Battery usage” were collected. For every application this feature shows how long it was actively used on screen and how long it was running in the background without the user engaging with it, but still consuming battery life. These durations were mostly available for the timeframe of the last ten days, on older iOS versions of the last seven days. Since this is a native iOS feature, users have no influence on the logging of their usage, ensuring objective and consistent data. The feature also shows grand total screen time, which was used as a reliability check when app usage was coded and summed up for analysis. In order to get an estimate of a subject’s average daily phone use, the total active screen time was divided by the timeframe indicated on the phone. To control for unusually long or short screen time at the time of data collection, participants had to report if their smartphone use was either unusually low, high or average within the last seven to ten days. If a subject indicated unusual usage and their self-reported usage differed from actual usage by more than 100%, participants were excluded from the analysis, which was not the case in our sample.

During data collection it became evident that some applications were used by almost all participants (e.g. WhatsApp, Facebook), while a vast amount of apps was installed only on few phones. Therefore, to allow for meaningful analyses screen time of apps that were used by less than a quarter of participants or had identical purposes (e.g. Safari and Chrome, Apple Mail and Yahoo Mail) was cumulated. This resulted in 11 distinct categories (see S1 Table for categorization). Additionally, in calculating net screen time we deducted screen time of applications related to music (e.g. Apple Music), TV (e.g. Netflix) and functionalities such as calling and GPS since these apps were characterized by passive usage, i.e. app running mostly in the background and/or requiring negligible interaction with the user. We assumed that inclusion of these usage patterns would distort the data; some participants had similar total screen time but in some cases this consisted mostly of social media use while in other instances the majority of active usage was due to GPS navigation.

#### Self-reported smartphone usage

Furthermore, to be able to compare these objective data to participants’ self-reports, four questions assessed phone-related behavior of the participants: 1) “how much time on average do you spend on app …”, 2) “how often do you usually post content or send messages on app … “, 3) “in which intervals do you normally check your phone for notifications” and 4) “after receiving a notification, how quickly do you click on it”.

#### Delay discounting

The tendency to prefer smaller immediate rewards over larger delayed rewards was assessed using a German translation of the 27-item Monetary Choice Questionnaire [15]. In this questionnaire participants have to repeatedly choose between a smaller reward available immediately (e.g. €15 today) or a larger reward available in the future (e.g. €35 in 13 days). All rewards are hypothetical and consist of small (e.g. €15), medium (e.g. €41) and large amounts of money (e.g. €80). The proportion of choices of the larger delayed reward (LDR) serves as a measure of impulsivity, i.e. the lower the proportion, the more impulsive the individual. The scale is widely used in the literature and provides similar results to more extended instruments [42]. Also, it is a robust finding that using hypothetical rather than real or potentially real rewards yields virtually the same results [43]. Furthermore, the proportion of LDR measure is a simple yet reliable and valid measure, which, unlike estimating the discounting rate using the method by Kirby et al. [15], does not assume hyperbolic discounting [44]. The responses to the MCQ were scored using automated scoring [45]. This tool also provides consistency scores to enable identification of a lack of attending to the questionnaire. None of our participants had consistency scores below 75%, indicating good quality of responses [46].

#### Reward valuation

In this study the neural process of reward valuation was operationalized as an individual’s responsiveness to rewards, which was elicited using the behavioral inhibition system/behavioral approach system (BIS/BAS) scales [47]. The scales measure an individual’s degree of behavioral inhibition and behavioral activation, the latter being subdivided into Drive, Reward Responsiveness and Fun Seeking. Rather than just using the Reward Responsiveness subscale, the full 24-item questionnaire with its mixed order of questions and filler items was administered to enable the best possible accuracy of results. The German version of the scales by Strobel et al. [48] were used for this study. While the full BAS scale showed acceptable internal consistency (α = 0.73), Cronbach’s alpha for the Reward Responsiveness subscale was low (α = 0.56) and therefore excluded from further analyses.

#### Cognitive control

We employed both a self-report as well as a behavioral measure of self-control, which has been shown to be closely linked to cognitive control processes [49]. For the self-report measure, we used the German adaptation by Bertrams and Dickhäuser [50] of Tangney et al.’s [51] brief self-control scale. Ample research has shown that the 13-item brief self-control scale yields reliable and valid results in assessing dispositional self-control capacity and performs similarly well compared to the less economical full 36-item version [51]. The internal consistency for this scale was acceptable (α = 0.74).

As a behavioral measure, we employed the Go/No-Go task as a test of response inhibition. In this task, participants have to respond as quickly as possible to rapidly-presented target (“Go”) cues shown on a computer screen. However, they have to withhold this response to non-target (“No-Go”) cues, which appear less frequently. Commission errors (i.e. responses to non-targets) are a measure of reduced impulse control. We implemented an adaptation of the task by Mostofsky et al. [52] in which green circles are used as target cues, to which participants have to respond by pressing the space bar, and red Xs as non-target cues; targets and non-targets were presented in a pseudorandom order. There were 127 Go-trials and 23 No-Go-trials per run; every 30 trials participants had a 10-second rest period to enable recovery of hemodynamic response. Participants completed two runs overall with a short self-determined rest period in between. Cues were shown for 200ms centered on a black screen. The intertrial-interval was 1,300ms during which a white fixation cross appeared in the center of the screen. The entire task took approx. 10 minutes to complete. In a pilot session, we determined that some participants did not exert full effort, e.g. by constantly pressing the space bar or by not responding at all. Therefore, to incentivize the task performance (measured by the error rate) we included in the instructions that the top 50% of participants would receive €2 for the task in addition to the base compensation, while the bottom 50% would receive no additional money. This resulted in having no shirkers in the main sessions.

#### Prospection

There are currently no established instruments that are suitable to measure an individual’s ability to imagine future experiences in healthy participants. Therefore, we used as a proxy the inclination to consider distant versus immediate consequences of potential behavior as measured by the consideration of future consequences (CFC) scale [53]. The CFC construct is conceptually similar to delay discounting and is likewise related to problematic behaviors [54], but may better reveal individual differences in the ability to project the self into the future as seen in day-to-day behavior [55]. The German translation of the 12-item questionnaire by Bruderer Enzler [56] was used. The internal consistency for this scale was good (α = 0.80).

### Procedure

In their invitations, participants were told that they needed to be iPhone users and bring their phone to the experiment. However, they were not informed about the experimental objective and methods to avoid participants from adapting their naturalistic behavior. The experiment was conducted in seven sessions. At the beginning of each session, participants were instructed about the tasks and in particular, the phone usage data collection and signed informed consent documents. They were, however, not allowed to access their phones until the end of the experiment. Participants then completed the Go/No-Go task followed by a simple five-minute long decision task, which was not relevant to this study. The experiment continued with the Monetary Choice Questionnaire, the BIS/BAS scale, the brief self-control scale and the consideration of future consequences scale. Lastly, phone use data was collected by taking photographs of the battery use screens on the participants’ phones. These data were entered into a spreadsheet after completion of each session. The order of tasks in our experiment was fixed throughout all sessions. On average, one session lasted about 50 minutes and participants received €16 in compensation. This study was approved by the German Association for Experimental Economic Research e.V. (approval no. xQ1XKNtp).

## Results

### Delay discounting and smartphone usage

The primary goal of this study was to determine if there was a positive relationship between actual smartphone usage and delay discounting. Indeed, we found a significant negative correlation between the proportion of choices of larger delayed rewards and net screen time (r = -0.25, p = 0.013), indicating that the more time is actively spent on a smartphone the less likely that individual is to wait for a larger award. The proportion of LDR choices was highly correlated (r = -0.98, p<0.001) with the natural logarithm of the discount parameter k according to Kirby et al. [15], indicating that the proportion measure was accurately assessing participants’ discounting of future rewards. In line with this, the natural logarithm of k was also correlated with net screen time (r = 0.21, p = 0.034). However, we did not find a significant relationship between total screen time and delay discounting. Table 1 shows bivariate correlations between the main variables in this study (see also S2 Table for descriptive statistics and S1 Appendix for additional correlations).

**Table 1:** Correlations between main variables.

Variable	1.	2.	3.	4.	5.	6.	7.	8.	9.	10.	11.
1. Net screen time	-										
2. Total screen time	0.94***	-									
3. Self-reported usage	0.56***	0.49***	-								
4. LDR proportion	-0.25*	-0.17	-0.23*	-							
5. ln overall k	0.21*	0.14	0.22*	-0.98***	-						
6. Self-control	-0.32**	-0.32**	-0.18	0.16	-0.14	-					
7. Consideration of future consequences	-0.17	-0.12	-0.12	0.07	-0.06	0.46**	-				
8. Response inhibition	0.00	-0.03	0.00	-0.19	0.22*	0.02	0.07	-			
9. Age	0.09	0.06	-0.13	0.05	-0.07	-0.05	0.06	-0.19	-		
10. Years of ownership	-0.04	-0.07	-0.08	0.11	-0.11	0.12	0.00	0.02	0.28	-	
11. Disposable income	-0.05	-0.07	0.06	-0.10	0.13	0.05	-0.09	0.05	0.14	0.12	-

*p < 0.05,

**p < 0.01,

*** *p* < 0.001.

Next, a regression analysis was performed to control for potential confounding variables in the relationship between net screen time and delay discounting (Table 2). In this analysis, we chose the LDR proportion (i.e. delay discounting) as the dependent variable without assuming a causal relationship between the two variables of interest. All assumptions for multiple regression were met. After controlling for demographic and psychological variables, net screen time was still a significant predictor of the LDR proportion (β = -0.24, p = 0.021), while all other independent variables were non-significant.

**Table 2:** Multiple regression analysis of predictors of LDR proportion.

Term	B	SE B	95% CI		β	t	p
			LL	UL			
Intercept	0.323	0.238	-0.149	0.795	0.000	1.359	0.178
Age	0.004	0.008	-0.011	0.020	0.079	0.571	0.569
Gender (Male)	0.035	0.018	-0.001	0.072	0.197	1.937	0.056
Education							
Hauptschulabschluss^a^	-0.195	0.153	-0.500	0.110	-0.281	-1.270	0.208
Fachabitur^b^	0.129	0.147	-0.164	0.422	0.186	0.872	0.385
Abitur^c^	0.046	0.057	-0.068	0.160	0.152	0.806	0.422
Bachelor	0.041	0.064	-0.086	0.169	0.116	0.646	0.520
Master/Diplom (Reference)							
Disposable income	0.000	0.000	0.000	0.000	-0.110	-1.065	0.290
Years of smartphone ownership	0.011	0.011	-0.012	0.033	0.102	0.954	0.343
Consideration of future consequences	0.000	0.003	-0.006	0.007	0.018	0.161	0.873
Self-control	0.001	0.003	-0.004	0.007	0.058	0.484	0.629
Response inhibition	-0.002	0.001	-0.004	0.001	-0.159	-1.542	0.127
Net screen time	-0.028	0.012	-0.053	-0.004	-0.243	-2.348	0.021

^a^ German school leaving certificate awarded after 9th grade.

^b^ German school certificate to enter University of Applied Sciences.

^c^ German High School Diploma.

Note: Effect coding was applied for categorical variables.

### Delay discounting and usage by app category

As screen time data by application was available, we next sought to determine which components of net screen time predict delay discounting by performing a multiple regression with the proportion of LDR choices as the dependent variable and application categories as independent variables. Controlling for demographic and psychological variables, social media and gaming apps turned out to be significant predictors of delay discounting (β = -0.27, p = 0.009 and β = -0.25, p = 0.024, respectively). All other regressors were not significant as can be seen in the regression results in Table 3. We also performed a robustness check on our app categorization; for a second regression we added participants’ YouTube screen time to the TV category instead of social media. Even with this adjustment social media and gaming apps remained significant, confirming these categories as predictors of delay discounting. The screen times of the most popular apps in our sample is shown in S3 Table.

**Table 3:** Multiple regression analysis of application categories.

Term	B	SE B	95% CI		β	t	p
			LL	UL			
Intercept	0.171	0.256	-0.338	0.680	0.000	0.668	0.506
Age	0.007	0.008	-0.009	0.024	0.133	0.884	0.379
Gender (Male)	0.028	0.018	-0.009	0.065	0.157	1.524	0.131
Education							
Hauptschulabschluss^a^	-0.263	0.151	-0.564	0.038	-0.380	-1.739	0.086
Fachabitur^b^	0.158	0.147	-0.135	0.450	0.228	1.073	0.286
Abitur^c^	0.072	0.058	-0.044	0.187	0.236	1.234	0.221
Bachelor	0.032	0.063	-0.094	0.158	0.090	0.505	0.615
Master/Diplom (Reference)							
Disposable income	0.000	0.000	0.000	0.000	-0.115	-1.108	0.271
Years of smartphone ownership	0.009	0.012	-0.014	0.033	0.090	0.807	0.422
Consideration of future consequences	0.000	0.003	-0.006	0.006	-0.005	-0.045	0.964
Self-control	0.003	0.003	-0.003	0.009	0.111	0.897	0.373
Response inhibition	-0.001	0.001	-0.003	0.001	-0.094	-0.881	0.381
Social Media	-0.044	0.017	-0.077	-0.011	-0.274	-2.676	0.009
Gaming	-0.087	0.038	-0.162	-0.012	-0.250	-2.300	0.024
Mail	-0.470	0.417	-1.299	0.360	-0.117	-1.126	0.263
Messenger	0.000	0.001	-0.002	0.002	0.005	0.051	0.960
Shopping	-0.034	0.147	-0.326	0.258	-0.024	-0.232	0.817
Browser	0.035	0.040	-0.045	0.115	0.090	0.864	0.390
Dating	0.051	0.068	-0.083	0.185	0.080	0.756	0.452
Other	0.127	0.071	-0.014	0.268	0.183	1.788	0.077

^a^ German school leaving certificate awarded after 9th grade.

^b^ German school certificate to enter University of Applied Sciences.

^c^ German High School Diploma.

Note: Effect coding was applied for categorical variables.

### Mediating role of self-control, response inhibition and consideration of future consequences

To investigate our second hypothesis, we initially examined the relationships of two psychological variables according to the model by Peters and Büchel [14] with actual smartphone usage and delay discounting. We found that self-control was negatively associated with net screen time (r = -0.32, p = 0.001), while all other relationships turned out to be non-significant at the 0.05-level (Table 1). We then performed mediation analyses using the PROCESS macro by Hayes [57]. This tool uses ordinary least squares regression, yielding path coefficients for total (i.e. between independent and dependent variable without mediator), direct (i.e. between independent and dependent variable with mediator), and indirect (i.e. through the mediator variable) effects. 5,000 bootstrap samples were constructed for each analysis to compute 95% confidence intervals and inferential statistics. Effects were deemed significantly different from zero when the confidence interval did not include zero. Three separate mediation analyses were performed to analyze whether self-control, response inhibition or consideration of future consequences could mediate the relationship between net screen time and delay discounting. We did not include reward responsiveness in the analyses, as the internal consistency of the scale was low. The indirect effects in all three models were non-significant (self-control B = -0.0032, CI [-0.0115, 0.0041]; response inhibition B = 0.0000, CI [-0.0051, 0.0042]; consideration of future consequences B = -0.0006, CI [-0.0049, 0.0034]). Compatibly, all direct effects remained significant, implying that neither self-control nor response inhibition nor consideration of future consequences played a mediating role in the relation between net screen time and delay discounting. Additional results of the mediation analyses are provided in S2 Appendix.

### Self-reported vs. actual usage

Lastly, to compare self-reported usage patterns to actual usage we examined the relationships between net screen time and self-reports with regard to usage time, posting and checking behavior as well as reaction to notifications. We also investigated the association of self-reports to delay discounting.

Net screen time was moderately associated with self-reported usage time (r = 0.56, p<0.001). In a head-to-head comparison of self-reported vs. actual screen time we found that 71% of participants overestimated and 17% underestimated their screen time. For only 12% of participants, actual screen time fell into the usage interval (e.g. “1.5 to 2 hours per day on average”) estimated by participants. Actual usage was also weakly related to checking behavior (r = 0.21, p = 0.035). When comparing self-reports to delay discounting, self-reported usage time and checking behavior were also associated with the LDR measure (r = -0.23, p = 0.022 and r = -0.21, p = 0.036, respectively). Reaction to notifications and posting behavior had no significant relationship neither to net screen time nor to the LDR measure.

## Discussion

In this study we set out to investigate the relationship between actual smartphone usage and personal dispositions. Being a correlate of a host of maladaptive behaviors, the variable of delay discounting was of major interest. Consistent with previous studies investigating the association between delay discounting and smartphone usage primarily based on self-reports [18, 19, 35], we found a positive relationship between actual smartphone usage and the discounting of future rewards. Our results suggest that as smartphone screen time increases, the tendency to choose smaller immediate rewards over larger delayed rewards increases as well, confirming our first hypothesis. This association provides further empirical evidence that smartphone usage compares to other maladaptive behaviors, such as smoking, gambling or drinking in the context of intertemporal choice.

We were also able to identify two application categories which predicted delay discounting, namely social media and gaming apps. This result seems intuitive since both types of apps offer gratification in the form of likes or entertaining content (social media) and rewards or bonuses (gaming). Recent research showing that behavior on social media conforms to the principles of reward learning [58] lends initial support to this interpretation. Both app categories were also used extensively (46 minutes and 35 minutes per day on average, respectively), while social media were much more present on participants’ phones than gaming apps (87% vs. 40% of phones). Interestingly, apps designed for shopping, a behavior shown to bear addiction potential [59], did not at all predict delay discounting. A possible explanation could be that online shopping was primarily done through other media, such as laptops or tablets—a hypothesis that is supported by the relatively short screen time of this app category (21 minutes per day on average). However, in interpreting these results it needs to be acknowledged that all application categories share similar mechanisms by sending notifications and quickly providing information, thereby involving gratification to some extent. More research is needed to uncover differences in the appeal of the various apps available to smartphone users.

When looking at the underlying mechanisms of delay discounting proposed by Peters and Büchel [14], we found that only self-control as assessed with the brief self-control scale was significantly correlated with net screen time; participants lower in self-control seemed to have greater difficulty in putting their phones aside than participants who reported to have higher self-control as observed in day-to-day behavior. This is in line with the finding of Wilmer and Chein [18] that heavier investment of time in a mobile device is related to weaker impulse control. However, our finding that the behavioral measure of self-control was not related to net screen time suggests that the cognitive process of response inhibition plays only a marginal role in how long a person engages with a smartphone. Interestingly, consideration of future consequences was neither associated with net screen time nor with delay discounting. On the one hand, this suggests that heavier smartphone users do not differ from lighter smartphone users in terms of the tendency to consider immediate vs. future outcomes of their day-to-day behaviors (as measured by the CFC scale). On the other hand, it seems that the CFC construct and delay discounting—despite their conceptual overlap—may not be used interchangeably when investigating their relationship with smartphone use.

We could not confirm our second hypothesis that the three psychological variables within the model of Peters and Büchel [14] mediate the relationship between smartphone screen time and delay discounting. This may indicate that smartphone usage has an idiosyncratic relationship with delay discounting, which cannot be explained with established concepts, namely self-control, response inhibition and consideration of future consequences. However, this preliminary conclusion needs further investigation, as we employed questionnaire-based and not neuroscientific methods (through which the model of interest has emerged) to assess the three psychological variables in this current study. Also, the possible mediating role of reward responsiveness has yet to be investigated. Moreover, for prospection we elicited a proxy variable, which might not have sufficient overlap with the concept proposed in the model of Peters and Büchel [14].

When comparing self-reports to actual usage data, we found that participants were able to estimate a general tendency of their screen time reasonably well. However, as expected these estimations were far from being accurate as indicated by the high percentage of under- and overestimations, suggesting that collecting actual data should be preferred whenever a high accuracy of data is required. This finding is in line with previous research highlighting the superiority of actual data in the context of smartphone usage [39].

These findings come with limitations, which may guide future research in the context of smartphone usage and its implications. First, we included only iPhone users in our sample, while users of other brands were not allowed to participate. While there is currently no reason to assume that smartphone usage differs systematically from iPhone to e.g. Samsung or Huawei phones, future studies should test our findings with other phone brands. Second, as restricted by the iOS feature participants’ smartphone application data of the 7–10 days leading into the experiment was taken as a basis for their average use. While we did consider participants’ comments about the “normality” of their latest usage patterns, using longer timeframes will result in more accurate data of user’s typical screen time. Third, some inaccuracy is inherent in the browser application data. A web browser allows for a multitude of uses, which includes most of the other app categories investigated in this study. As we did not collect browsing history data, we were not able to determine what exactly our participants used their browser for, contributing to noisiness of the usage data. Fourth, the Monetary Choice Questionnaire employed in our study has several drawbacks. While it is very efficient, it is not the most sensitive instrument to assess delay discounting [60]. For instance, within the scale the smaller sooner option is always set to the present, thereby omitting intertemporal choices in which both rewards are available at different points in the future. This bears the risk of overweighing present bias in measuring delay discounting [61]. Furthermore, unusual discounters (i.e. participants with either negative or extremely high discount rates) cannot be captured with the Monetary Choice Questionnaire, as the scale only permits nine discrete discount rates between 0.00016 and 0.25 [62]. Future studies could employ e.g. computer-based, adjusting delay discounting tasks or even more general measures of time inconsistency as recently proposed e.g. by Rohde [63]. Lastly, all relationships reported in this study are correlational in nature, meaning that no inferences on causality can be made. Using our main finding as an example, enduring smartphone usage may cause an individual to become a more impulsive decision-maker over time. However, it is also possible that individual differences in the preference for immediate rewards result in investing more time in smartphone engagement. The latter relationship currently seems more likely, given the initial finding of Hadar et al. [35] that a three-month smartphone exposure did not cause any changes in impulsive decision-making, but more longitudinal research is needed.

Given the ever-growing role smartphones play in people’s daily lives and the implied risk of overuse, it is crucial to understand individual differences which relate to smartphone usage. In this study, we provided further evidence for a behavioral similarity between smartphone usage and other maladaptive behaviors. Our findings suggest that especially heavy social media users and gamers should be mindful of their tendency to be drawn to smaller, immediate rewards. Alternatively, people who are already aware of their impulsive decision-making may benefit from the knowledge of their increased risk of overusing smartphones. These conclusions contribute to the view that smartphone use should not be underestimated but researched carefully to guide policy makers in shaping prudent use of this omnipresent technology.

## Supporting information

S1 Table Categorization of applications. (DOCX)

S2 Table Descriptive statistics for the main measures. Participants spent on average 3 hours and 12 minutes, at least 12 minutes and at most 8 hours and 24 minutes per day interacting with their smartphone. On average, the larger delayed reward was chosen 45% (minimum 7%, maximum 100%) of the time.
(DOCX)

S3 Table Top 5 applications according to net screen time. Instagram was present almost on 75% of phones and had the highest average daily screen time of 46 minutes. The average net screen time per day is calculated over the number of phones on which the respective app was installed, so as to account for the prevalence of apps. 64% of participants used the YouTube app and spent on average 39 minutes per day engaging with it. This was followed by WhatsApp, which was installed by almost all participants, with an average screen time of 37 minutes.
(DOCX)

S1 Appendix Additional correlations. (DOCX)

S2 Appendix Mediation diagrams. (DOCX)

S1 Dataset Raw data from the questionnaire and screen time data. (XLSX)

S2 Dataset Raw screen time data. (XLSX)
