# Supplementary material for: Likes and impulsivity: Investigating the relationship between actual smartphone use and delay discounting
Source: PLoS One. 2020 Nov 18;15(11):e0241383. doi: 10.1371/journal.pone.0241383 (PMC7673521; doi:10.1371/journal.pone.0241383)
Supplement: S1 Table — (DOCX) [file pone.0241383.s001.docx]

**S1 Table. Categorization of applications.**

| **Category** | **Examples** |
| --- | --- |
| Social Media | Facebook, Instagram, Twitter, YouTube |
| Messenger | WhatsApp, Facebook Messenger, Snapchat |
| Dating | Tinder, Bumble |
| Browser | Safari, Chrome, Ecosia |
| Games | Clash Royale, PUBG Mobile, Pokémon GO |
| Shopping | Amazon, Zalando, Kleiderkreisel |
| Music/Podcasts | Apple Music, Spotify, Apple Podcasts |
| Mail | Apple Mail, Gmail, Yahoo Mail |
| TV | Sky, Netflix, Primevideo, DAZN |
| Other | Dict.cc, Sparkasse, Photos |
| Functionalities | Camera, Settings, Call, Google Maps |
