# Supplementary material for: Likes and impulsivity: Investigating the relationship between actual smartphone use and delay discounting
Source: PLoS One. 2020 Nov 18;15(11):e0241383. doi: 10.1371/journal.pone.0241383 (PMC7673521; doi:10.1371/journal.pone.0241383)
Supplement: S2 Table — Participants spent on average 3 hours and 12 minutes, at least 12 minutes and at most 8 hours and 24 minutes per day interacting with their smartphone. On average, the larger delayed reward was chosen 45% (minimum 7%, maximum 100%) of the time. (DOCX) [file pone.0241383.s002.docx]

**S2 Table. Descriptive statistics for the main measures.**

|  | **Mean** | **Std. dev.** | **Min** | **Max** |
| --- | --- | --- | --- | --- |
| **Average net screen time per day [h]** | 3.2 | 1.5 | 0.2 | 8.4 |
| **Media Multitasking** | 2.3 | 0.5 | 1.2 | 3.4 |
| **Proportion of LDR [%]** | 45 | 18 | 7 | 1 |
| **Reward sensitivity** | 16.7 | 2.1 | 10 | 20 |
| **Go/No-Go commission error [%]** | 20.3 | 15.8 | 0 | 68.5 |
| **Self-control** | 39.9 | 7.4 | 24 | 61 |
| **Consideration of future consequences** | 43.9 | 6.72 | 27.96 | 56 |
