# Supplementary material for: Likes and impulsivity: Investigating the relationship between actual smartphone use and delay discounting
Source: PLoS One. 2020 Nov 18;15(11):e0241383. doi: 10.1371/journal.pone.0241383 (PMC7673521; doi:10.1371/journal.pone.0241383)
Supplement: S3 Table — Instagram was present almost on 75% of phones and had the highest average daily screen time of 46 minutes. The average net screen time per day is calculated over the number of phones on which the respective app was installed, so as to account for the prevalence of apps. 64% of participants used the YouTube app and spent on average 39 minutes per day engaging with it. This was followed by WhatsApp, which was installed by almost all participants, with an average screen time of 37 minutes. (DOCX) [file pone.0241383.s003.docx]

**S3 Table. Top 5 applications according to net screen time.**

| **Application** | **Average net screen time per day [min.]** | **Occurrence [#]** |
| --- | --- | --- |
| **Instagram** | 46 | 76 |
| **YouTube** | 39 | 65 |
| **WhatsApp** | 37 | 99 |
| **Games** | 35 | 41 |
| **Browser** | 27 | 101 |
