# Supplementary material for: Likes and impulsivity: Investigating the relationship between actual smartphone use and delay discounting
Source: PLoS One. 2020 Nov 18;15(11):e0241383. doi: 10.1371/journal.pone.0241383 (PMC7673521; doi:10.1371/journal.pone.0241383)
Supplement: S1 Appendix — (DOCX) [file pone.0241383.s004.docx]

**S1 Appendix. Additional correlations of screen time and delay discounting.**

| Variable | 1. | 2. | 3. | 4. | 5. | 6. |
| --- | --- | --- | --- | --- | --- | --- |
| 1. LDR proportion | - |  |  |  |  |  |
| 1. ln overall k | -0.98*** | - |  |  |  |  |
| 1. Net of music, TV and functionalities | -0.25* | 0.21* | - |  |  |  |
| 1. Net of music | -0.16 | 0.13 | 0.98*** | - |  |  |
| 1. Net of TV | -0.23* | 0.19 | 0.94*** | 0.96*** | - |  |
| 1. Net of functionalities | -0.20* | 0.16 | 0.95*** | 0.98*** | 0.94*** | - |

^*^*p* < 0.05, ^**^*p* < 0.01, *** *p* < 0.001

The table shows various alternative calculations of net screen time and their correlations with both parameters of delay discounting. When only screen time of TV apps is deducted from total screen time, this variant is significantly correlated with the LDR proportion (r=-0.23, p < 0.05). When only screen time of functionalities (settings, GPS etc.) is subtracted and TV and music apps are included, there is still a significant correlation with the LDR proportion (-0.20, p < 0.05). The latter indicates that the main result of this paper is fairly robust, given the overall weak association between screen time and delay discounting.
