# Supplementary material for: Likes and impulsivity: Investigating the relationship between actual smartphone use and delay discounting
Source: PLoS One. 2020 Nov 18;15(11):e0241383. doi: 10.1371/journal.pone.0241383 (PMC7673521; doi:10.1371/journal.pone.0241383)
Supplement: S2 Appendix — (DOCX) [file pone.0241383.s005.docx]

**S2 Appendix. Mediation diagrams.**

1. Self-control as mediator

Self-control

Delay discounting

Net screen time

a = -1.517**

b = 0.002

c = -0.029*
c‘ = -0.026*

^*^*p* < 0.05, ^**^*p* < 0.01

2. Response inhibition as mediator

b = -0.002

a = -0.002

Delay discounting

Response inhibition

Net screen time

c = -0.029*
c‘ = -0.029*

^*^*p* < 0.05, ^**^*p* < 0.01

3. Consideration of future consequences as mediator

Delay discounting

Net screen time

Consideration of fu-ture consequences

a = -0.725

b = 0.001

c = -0.029*
c‘ = -0.028*

^*^*p* < 0.05, ^**^*p* < 0.01
